# Stable *Plasmodium falciparum* merozoite surface protein-1 allelic diversity despite decreasing parasitaemia in children with multiple malaria infections

**DOI:** 10.1186/s12936-025-05378-7

**Published:** 2025-04-28

**Authors:** Reuben M. Yaa, Kelvin M. Kimenyi, Henry Antonio Palasciano, George Obiero, L. Isabella Ochola-Oyier

**Affiliations:** 1 Department of Biochemistry, University of Nairobi, Nairobi, Kenya; 2 UK Dementia Research Institute, Imperial College London, London, UK; 3 Department of Brain Sciences, Imperial College London, London, UK; 4 KEMRI-Wellcome Trust Research Programme, Kilifi, Kenya; 5 Department of Mathematics, Imperial College London, London, UK

**Keywords:** Malaria, *Pf*msp1, Diversity, Microhaplotype, Parasitaemia

## Abstract

**Background:**

Individuals experiencing recurrent malaria infections encounter a variety of alleles with each new infection. This ongoing allelic diversity influences the development of naturally acquired immunity and it can inform vaccine efficacy. To investigate the diversity and infection variability, *Plasmodium falciparum* merozoite surface protein 1 (*Pf*MSP1), a crucial protein for parasite invasion and immune response, was assessed in parasites isolated from children in the Junju cohort, Kilifi County, who experienced at least 10 febrile malaria episodes over a span of 5 years.

**Methods:**

*Pfmsp1* C-terminal region (*Pf*msp1_19_) was genotyped using PCR followed by capillary sequencing in blood samples collected from the children. Sequenced reads were trimmed and aligned to the *P. falciparum* 3D7 reference genome. Single nucleotide polymorphisms in the *Pf*msp1_19_ region were identified from the alignment and grouped to distinct microhaplotypes whose changing frequency over time were examined across the multiple infection episodes. In addition, the variability of infections in the population was assessed using nucleotide and haplotype diversity indices.

**Results:**

A total of eleven microhaplotypes were observed across all malaria episodes. There were 3 prevalent microhaplotypes, E-KSNG-L, Q-KSNG-L, and Q-KSNG-F in the population. Conversely, microhaplotypes such as Q-KNNG-L, E-KSSR-L, E-KNNG-L, E-KSSG-L, E-TSSR-L (3D7), Q-TSSR-L, E-TSSG-L, and E-KSNG-F were rare and maintained at low frequencies. High allelic replacements were observed, however some individuals experienced consecutive re-infections with the same microhaplotype. Notably, *Pf*MSP1_19_ allelic diversity as measured by haplotype diversity was stable, while nucleotide diversity decreased over time with decreasing parasitemia. Parasite *Pf*MSP1_19_ allelic diversity remained stable over the multiple malaria episodes, despite declining parasitaemia levels. In addition, there are reveal dynamic *Pf*MSP1_19_ allelic replacements across parasite infection episodes.

**Conclusions:**

Allelic diversity was stable over time in individuals, based on this limited polymorphic region and small sample size, suggesting that there are no significant shifts in allele frequencies or replacements due to alleles being maintained under balancing selection. The dominant alleles in the population are those frequently observed in these children with multiple malaria episodes, further suggesting that early exposure to dominant alleles does not shift their frequency in the population or prevent repeat infection with the same alleles in subsequent infections. However, a blood stage merozoite vaccine is likely to require a multi-allelic formulation.

**Supplementary Information:**

The online version contains supplementary material available at 10.1186/s12936-025-05378-7.

## Background

In an attempt to evade host immunity during infection, *Plasmodium falciparum* parasites regularly replace merozoite antigen epitope conformations and thus have the option to use alternative invasion pathways, disrupting complement activation [1–4]. At the genetic level, the changes may arise from point mutations leading to single nucleotide polymorphisms (SNPs), insertions/deletions of one or more base residues as well as meiotic recombination events of parental alleles, generating newer progeny [5, 6]. This is further maintained by the phenomenon of balancing selection. This process stabilizes the polymorphic circulation of immune targeted antigens resulting in multi-allelic circulation of these genes especially in malaria-endemic regions [7]. Effects of balancing selection have been shown before in merozoite surface protein (MSP) 1 [8], apical membrane antigen-1 (ama1) [9], MSP3 [10], erythrocyte binding antigen-175 (EBA-175) [11], MSP Duffy binding Ligand-1 and 2 (MSPDBL1 and MSPDBL2) [12, 13], reticulocyte binding homologues-2 (Rh2) [14, 15] and Rh5 [16].

Merozoite antigen alleles are maintained by frequency-dependent immune selection which shifts allele frequencies over time. This is because the rare alleles are observed less but later rise to high frequency [17]. This pattern of selection supports allele-specific immunity, which has repeatedly been shown to reduce overall vaccine efficacies that are based on low frequency or single alleles in different malaria endemic settings [18–20]. Of interest is *P. falciparum* MSP1, which has been advanced over time as a vaccine candidate, and it is a suitable marker for genotyping parasite populations in anti-malarial efficacy studies and clinical trials [21]. However, allelic replacements are associated with reduced efficacies on vaccine formulations targeting this antigen [22–25].

*Pf*MSP1 is a predominant antigen on the surface of the asexual blood stage of the parasite that plays an imperative role in erythrocyte invasion to cause malaria clinical symptoms. It is synthesized as a large precursor during schizogony and subsequently processed via proteolytic cleavage into 5 fragments of which the smallest is a 19kDa fragment (*Pf*MSP1_19_). This fragment has two epidermal growth factor (EGF) domains, one located at the C-terminal and another at the N-terminal ends. The C-terminal interacts with band 3, the erythrocyte receptor, to facilitate parasite erythrocyte invasion [26]. Inside the erythrocyte, the parasite multiplies and later egresses into the bloodstream following the rupture of the erythrocyte, a process in which *Pf*MSP1_19_ is also involved [27]. During egress, subtilisin-like (SUB1) parasite serine protease modifies the structure of *Pf*MSP1 to bind spectrin, a component of the host erythrocyte cytoskeleton to facilitate egress [28]. Genetic diversity studies of *Pf*MSP1 have highlighted that fewer polymorphisms are located at the 19kDa fragment than the rest of the protein, a total of 6 polymorphic loci [29]. Probably, because of its direct proximal interaction with its receptor. The 19kDa fragment is easily accessible to the host immune system as evidenced by merozoite invasion and parasite growth inhibition with antibodies in in vitro and mice experiments [22, 26, 29]. The fragment elicits both humoral and cell mediated immune responses during exposure to natural infections [25, 30], particularly to the polymorphic amino acids at the second EGF-like domain [31].

Allelic diversity of *Pf*MSP1 at the C-terminal region has been shown previously in malaria endemic regions such as Kenya, Tanzania and Uganda [31–34]. Similarly, significant epitope diversity through immune assays [17] have been demonstrated before in longitudinal studies. Though *Pf*MSP1 allelic diversity and patterns have previously been investigated in different malaria endemic regions, it has not been assessed in recurrent multiple infections in moderate to high malaria transmission regions. To achieve this, allelic replacements and the distribution of C-terminal *Pfmsp1* microhaplotypes were determined over time in multiple infections to describe *P. falciparum* infection diversity. Interrogating *Pfmsp1* microhaplotypes in individual infections over time will shed light on parasite genetic diversity in individual infections, providing a background of allelic replacement based on a region of the *msp1* gene with limited polymorphisms.

## Methods

### Study design

The study utilized samples from a larger cohort in an integrated study on natural immunity to malaria established in 2005 in Junju, Kilifi County, Kenya, where malaria transmission was high [35]. Sample collection was conducted under institutional ethical review (SERU 3149) with sampling done from 2008 to 2013. A blood sample was obtained from every participant upon confirmation of a febrile malaria episode followed by artemether-lumefantrine first-line treatment. From the blood samples malaria parasitaemia load was estimated using microscopy. In this study, children who had > 2 infections per year [36] resulting to 33 children (comprising 19 males and 14 females) were selected, each with at least a minimum of 10 malaria episodes over the 5-year sampling period. All together this resulted in a total of 426 blood samples. The blood samples were used to evaluate the *Pf*MSP1 C-terminal coding region from the parasite isolates.

### DNA extraction, PCR and sequencing

Total genomic DNA from blood samples were extracted using the QIAamp Blood Mini Kit (Qiagen). The 272bp *Pf*msp1 19kDa coding region was amplified by Polymerase Chain Reaction (PCR) using High Fidelity Taq polymerase (Sigma Aldrich, cat. no:11732641001) with *Pf*msp1_19_-F 5′-CAATGCGTAAAAAAACAATGTCC-3′ and *Pf*msp1_19_-R 5′-TTAGAGGAACTGCAGAAAATACCA-3′ specific primers pairs on cycling conditions as follows: 1 cycle at 94 °C for 2 min, 9 cycles of 94 °C for 30 s, 44 °C for 30 s, 72 °C for 2 min, 24 cycles of 94 °C for 30 s, 44 °C for 30 s, 72 °C for 2 min + 5 s per cycle and a final step of 72 °C for 2 min. The amplified PCR products were separated by 2% (w/v) agarose gel electrophoresis in a buffer composed of 40 mM Tris, 1 mM EDTA and 20 mM Acetic acid (TAE), pH 8.2, for 40 min at 100 V. PCR products were visualized using 1% SYBR (v/v) safe stained agarose gels and cleaned using ethanol precipitation. Sequencing templates were prepared using BigDye^™^ Terminator v3.1 cycle sequencing kit. A volume of 3 μl of the purified products was resuspended with, 4 μl BigDye^™^ Terminator 3.1 ready reaction mix, 1 μl of 10 μM of *Pf*msp1_19_-F primer used during fragment isolation and 3 μl of deionized water to a total reaction volume of 10 μl in 96 well plate. Cycle sequencing of the amplicons was done using PCR as follows: 96 °C for 1 min, 25 cycles of 96 °C for 10 s, 50 °C with + 1 °C/second for 5 s and 60 °C for 4 min. Sequencing reactions were purified using ethanol/EDTA precipitation and reactions resuspended in Hi-Di^™^ formamide. The plates were then analysed using capillary electrophoresis on ABI 3500XL Genetic Analyzer outsourced from Inqaba Biotechnical Industries (Pty), South Africa. Sequences were assembled, trimmed and edited using Sequencher^®^ 5.3 DNA analysis software (Gene Codes Corporations, Ann Arbo, MI USA) and CLC sequence viewer version 7(QIAGEN). DNA sequence data and corresponding translated protein were aligned to *P. falciparum* 3D7 *msp1* (PF3D7_0930300) msp1 (PF3D7_0930300) reference sequence, ASM276v2, using the MUSCLE alignment algorithm in the MEGA 11 program [37, 38]. The sequences were deposited in the GenBank NIH genetic sequence database under accession numbers (OQ821998–OQ822147).

### Data processing and statistical analyses

After standardizing the sequences to the same length (234bp) and excluding short sequences that did not cover the segregating sites in either orientation, sequences were clustered using USEARCH v11 software [39] and Phyclust R package [40] to identify microhaplotypes. This was followed by determining microhaplotype frequencies. Phyclust applies grouping of microhaplotypes and categorizes those to be retained above a cut-off point which is an optimal balance between the sample size, microhaplotype number and frequencies [40]. Microhaplotype sequences were extracted as an alignment and transformed into a DNAbin object using ape R package [41]. The object was transformed into a hamming distance matrix by measuring pairwise distances of corresponding residues between microhaplotype pairs, while counting differences between them and storing this on a symmetric matrix which was visualized as a heatmap [42].

Temporal population infection variability analysis was conducted to examine the genetic diversity of the *Pfmsp1* locus using haplotype and nucleotide diversity indices. Microhaplotypes were assigned back to the patients to assess patient-microhaplotype distribution and proportions. The time between infections for individuals was determined by calculating time elapsed between successive infections.

To determine parasitaemia and microhaplotype dynamics over time, the outcome of *Pfmsp1*_*19*_ PCR on all the samples and genetic diversity of the microhaplotype was correlated using the Spearman rank method with malaria parasitaemia. To do so, we only used data corresponding to the first 14 episodes, since the amount of data collected beyond this episode was insufficient to appropriately carry out this analysis. In addition, microhaplotypes that did not have respective parasitaemia data were excluded from the analysis. The samples were grouped based on *Pfmsp1*_*19*_ PCR amplification status as either amplicon present or absent. Parasitaemia was determined by microscopic examination of blood films of *P. falciparum* parasites by counting the number of parasites/200 white blood cells [35] (Supplementary Table 1). The difference in parasitaemia between the two groups was compared using the Wilcoxon rank-sum test. In addition, the correlation between parasitaemia and microhaplotype diversity was assessed over the multiple infection time points.

## Results

### Microhaplotype classes and associated patterns in the population

**Fig. 1:**
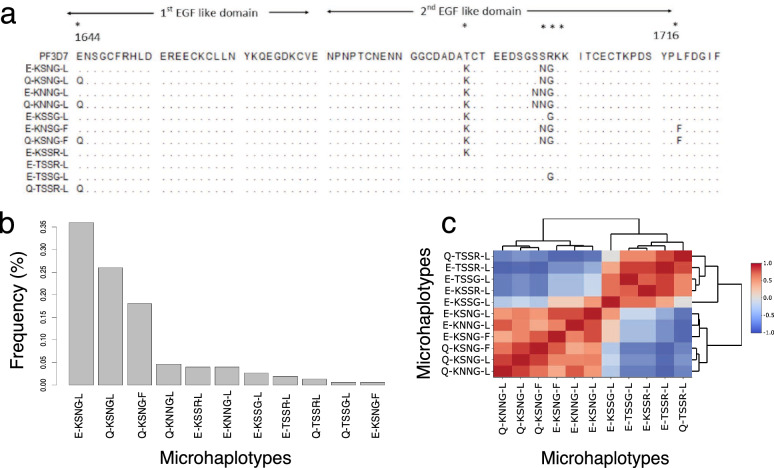
Dynamics of *P. falciparum* msp1_19_ microhaplotypes. **A** Amino- acid sequence alignment of 11 identified microhaplotypes. Polymorphic sites are shown with an asterisk (*). The nucleotide positions relative to the start position of the *Pfmsp1* gene are shown below the asterisk. The dots in the alignment indicate the position corresponding to *P. falciparum 3D7* with identical amino acid sequences. The epidermal growth factor (EGF)-like domains 1 and 2 are shown by arrows. The first polymorphism is located in the first EGF-like domain, whereas the second to the fifth polymorphism are located in the second EGF-like domain. **B** Microhaplotypes sorted by their abundance in the population. **C** Microhaplotypes clustered to groups based on the number of nucleotide differences between haplotypes. The dendrogram on the sides of the heatmap visually represents the relatedness of the microhaplotypes. In this context, the branches indicate distinct clusters formed through hierarchical clustering, highlighting groups of haplotypes with similar characteristic

The *Pfmsp1*_*19*_ fragment was genotyped from blood samples of 33 children (19 males and 14 females) with infections spread between 10 and 24 multiple episodes totaling to 426 infections. On recruitment, the average age of the participants was (5.5 ± SD 1.8 years), and at the end of study was (10.30 ± SD 1.8 years). A total of 64.8% (276/426) of the samples yielded *Pfmsp1*_*19*_ amplicons, out of this, data for total of 65.2% (180/276) was obtained in which 54.3% (150/276) generated the full length (234bp) *Pf*msp1_19_ C-terminal contigs that were used for the microhaplotype analysis. The median parasitemia was significantly higher in the samples from which *Pfmsp1*_*19*_ amplicons were generated, 160,000 parasites/µl (interquartile range (IQR) = 245,740) compared to those where no *Pfmsp1*_*19*_ amplicons were generated, 15,800 parasites/µl (IQR = 113,640) (P < 0.0001). Throughout the entire period, an average of 5 samples (± SD 2.678) were genotyped per individual, which yielded a total of 150 sequences. Six distinct nucleotide polymorphisms were identified at positions 4990, 5132, 5157, 5159, 5161, and 5206 in relation to *Pfmsp1* reference gene coordinates. The nucleotide substitutions in the polymorphic sites resulted in non-synonymous amino acid substitutions at codons 1644, 1691, 1699, 1700, 1701 and 1716 yielding a total of 11 microhaplotypes (Fig. 1A), all of which have been reported in previous studies [21, 43]. Microhaplotypes E-KSNG-L, Q-KSNG-L and Q-KSNG-F corresponding to FUP-Uganda PA, FVO Wellcome and Thai (T807) strains, respectively, were the dominant microhaplotypes circulating in the population with proportion frequencies of 36% (54/150), 26% (39/150) and 18% (27/150), respectively. The remaining haplotypes which included Q-KNNG-L, E-KSSR-L and E-KNNG-L, E-KSSG-L, E-TSSR-L (3D7), Q-TSSR-L, E-TSSG-L and E-KSNG-F were circulating with frequencies of 4% and below and were considered as rare microhaplotypes (Fig. 1B).

**Fig. 2:**
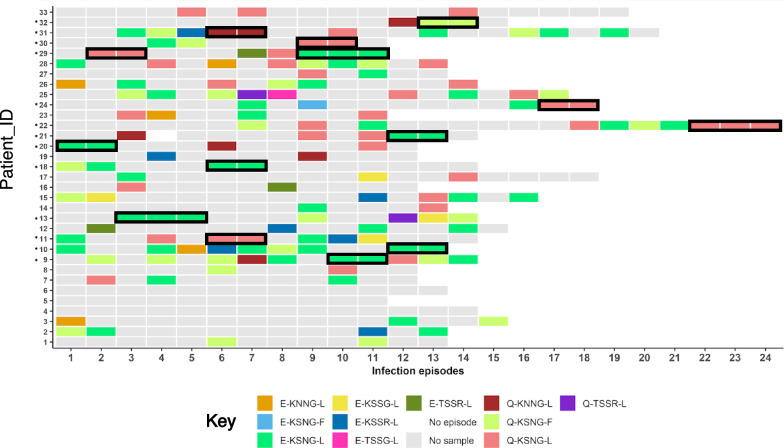
Microhaplotype patterns across the infections. The distribution of microhaplotypes in each patient across the malaria episodes. No sample—Samples not retrieved from the biobank, samples not genotyped or sequenced. No episode—No malaria episode was reported. Patient_IDs with asterisks represent cases that had 2–3 similar consecutive microhaplotypes outlined in black. Patients_IDs 4, 5 and 6 did not yield sequenced data and were excluded from the analysis

At least 7 of the 33 patients exhibited infections with several different microhaplotypes ranging from 7 to 10 over the entire infection period (Fig. 2A, Supplementary Fig. 1 A). All patients were infected with at least one or more of the predominant microhaplotypes. Specifically, 72.7% (24/33) were infected with the E-KSNG-L predominant microhaplotype. This microhaplotype ranged from 22.2 to 75% per patient in relation to all genotyped re-infections per individual (Supplementary Fig. 1B).

The microhaplotype hamming distance matrix classified the microhaplotypes into 3 groups based on Pearson correlation measures (Fig. 1C). The first larger group was composed of E-KSNG-L, Q-KSNG-L and Q-KSNG-F, the prevalent haplotypes, and E-KNNG-L, E-KSNG-F and Q-KNNG-L which were rare circulating microhaplotypes. The second group was made up of a single microhaplotype, E-KSSG-L, and the third group included E-KSSR-L, E-TSSG-L, E-TSST-L and Q-TSSR-L microhaplotypes which were also rare circulating microhaplotypes (Fig. 1C**)**.

### Microhaplotype dynamics over time and across infections

The pattern of the microhaplotypes were examined to investigate the allelic replacements of the C-terminal of *Pf*msp1_19_ over the course of multiple infections. At an individual level, there were notably high random allelic replacements between re-infections. However, 3 children (Patient_IDs 4, 5 and 6) did not have any sequenced data over the infection periods and were excluded from the analysis (Fig. 2). At least 39.4% (13/33) of the patients were consecutively re-infected with the same microhaplotype of the prevalent alleles either 2 or 3 times across the infection (Fig. 2).

**Fig. 3:**
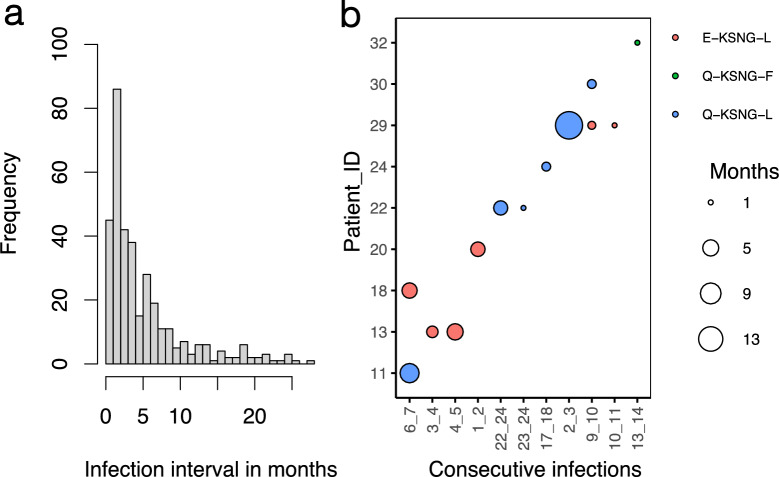
Distribution of infection intervals. A Frequency histogram of the distribution of the time interval between infections in months for all infections with genotype data. **B** Interval in months for individuals with consecutive infections of the same microhaplotype. The size of the circles depicts the number of months between infections with the same microhaplotype depicted by the color of the circle

Except for one individual (1 out 14) the consecutive re-infections occurred within a one-year timeframe (Figs. 2, 3B). Remarkably, all individuals experiencing consecutive re-infections with the same microhaplotype, apart from one (1/13), showed no recurrence of those specific microhaplotypes in subsequent parasite infections (Fig. 2). The average interval between infections for the entire period was 5.0 months, ranging from 1 and half weeks to around 25 months (Fig. 3A).

### Correlation of parasitaemia, genotyping and microhaplotypes

**Fig. 4:**
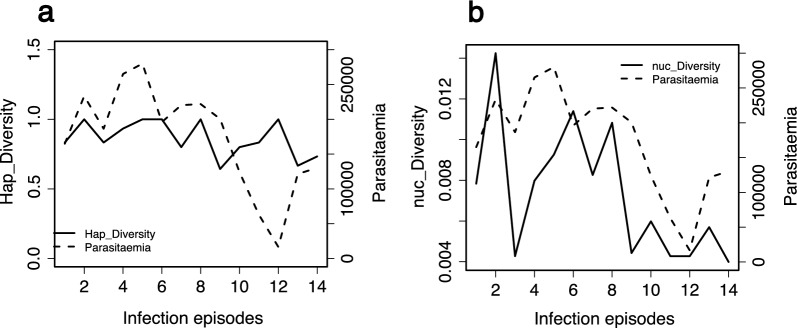
Parasitaemia correlations with measure of genetic diversity across infection episodes. **A** Haplotype diversity (hap_diversity) fluctuates within a small range (between 0.6 and 1) across infection episodes as parasitaemia (parasites/µl) reduces, the correlation between haplotype diversity and parasitemia was low, 0.37. **B** Nucleotide diversity (nuc_diversity) reduces concurrently across infection episodes with parasitaemia with a correlation of 0.7

The change in parasitaemia levels was examined over time and correlated with microhaplotype and nucleotide diversities across the infection episodes. During the early and middle stage infection episodes (< 8 episodes) parasitaemia levels were notably high. However, as the infections progressed towards the later (> 8) episodes, parasitaemia levels exhibited a decreasing trend. The genetic diversity of the locus fluctuated at the nucleotide level across the infection episodes, whereas the microhaplotype diversity remained stable (Fig. 4). The positive correlation between parasitaemia and nucleotide diversity was stronger (correlation coefficient, 0.7) than that for microhaplotype diversity, 0.37.

## Discussion

Host immune responses continuously shape merozoite antigen diversity [7, 44–46] by shifting allele frequencies and maintaining the presence of rare alleles. The diversity of *Pfmsp1* in parasite isolates from children with multiple malaria infections (a proxy of developing immunity) was used as a window into assessing the perturbation on merozoite antigen allele diversity. Haplotype diversity captured linked genetic regions in *Pfmsp1* and it was maintained even as parasitaemia levels declined over multiple malaria infections in an individual.

At a population level, a similar presence and prevalence of the common *Pf*MSP1_19_ microhaplotypes have been observed across sub-Saharan Africa including in the Coast of Kenya, Western Kenya, Republic of Congo, Uganda, Tanzania, Mali and Burkina Faso [7, 21, 34, 47, 48], suggesting that in moderate to high transmission, *P. falciparum* populations maintain a complex infection pattern that supports out-breeding while preserving genetic diversity.

It is expected that following multiple exposures to a single allele, immunity develops and reduces its frequency in subsequent infections. However, repeat infections with the prevalent alleles circulating in the population was common, with over a third of the children showing consecutive infections with the same allele while 6 children did not show a repeat infection with the same allele. The limitation in these 6 children is the low number of genotyped samples. Importantly, these findings highlight the complexity of the parasite's genetic diversity that needs to be determined in the light of other polymorphic antigens. Furthermore, this region of the *msp1* antigen is limited in genetic diversity, and the capillary sequencing method, limits the demonstration of distinct allelic changes with high resolution. Other genetically diverse antigens may show differences between each infection, such as *ama1* or block 2 of *msp1* and block 3 of *msp2*. Though there was a reduction in parasitaemia in later infections haplotype diversity remained stable. The control of parasitaemia following several malaria infections is similar to previous findings in Uganda that observed lower parasite densities with increasing age and in high malaria transmission areas [49]. Thus, emphasizing the difference in the immunity that controls parasitaemia and that which could lead to sterile immunity. This latter process is not achieved for malaria, a possible reason as indicated by this data is the re-infection of individuals with the same prevalent alleles. The reinfection with the same alleles may be due to an ineffective immune response that is not protective, akin to the original antigenic sin hypothesis [50]. The re-infections with the same allele allows the maintenance of their high prevalence in the population and thus the genetic diversity of the infections is unaltered. The high haplotype diversity was sustained, while the nucleotide diversity in contrast dropped with the parasitaemia levels. The nucleotide diversity is likely to reduce as re-infections occur with the same allele, the average nucleotide differences between sequences across the population will thus reduce. However, it will not alter the overall haplotype diversity the probability that two randomly sampled alleles are different in the population. Furthermore, this population is unique since these children are a subset of those from a previous study [51] who were uncharacteristically infected several times over 5 years with malaria. They were shown to have a modified immune system of high immune activation and inflammation, TNF, IL-6, IL-10 and cell populations such as γδ T cells were significantly higher in children with > 8 malaria infections compared to those with < 5 infections [51]. This skewed cytokine profile may act in a way that the inflammatory immune response to some extent clears parasites controlling parasitaemia.

Of additional interest, was the time between infections that was on average 5 months for the recurrent allelic infections, corroborating that immunity to malaria is not sterile and re-infections are common following waning of immunity or the high susceptibility of these children to re-infection which occurred after 5 months. Furthermore, there was no skew to specific alleles in these infections and the dominant 3 alleles were maintained genetic diversity in the population, providing a challenge for blood stage malaria vaccine design that will likely require a multi-allele formulation.

Interpretation of results is subject to some limitations. First, other more polymorphic merozoite genes should be included to determine whether they complement these findings. Secondly, the analysis only identified the dominant genotypes, and the polyclonality of the infection was not determined. Previous data from this cohort has shown that between paired infections, in the same individual utilizing more polymorphic antigens, msp2 block 3 capillary fragment analysis and ama1 amplicon deep sequencing, that the infections have different haplotypes [52, 53]. Finally, the biological consequences of these variants across the infections was not accessed to define the immunological impact. Functional immunological validation experiments are required to determine whether the absence of consecutive same variant infections were the result of allelic-specific or allele-transcending immune responses.

## Conclusion

Parasite *Pfmsp1*_*19*_ allelic diversity remains stable over the multiple malaria episodes despite declining parasitaemia levels, possibly reflecting the development of anti-parasite immunity. While shifts in alleles between infections appear to be random, the re-infections with the dominant alleles suggests that immunity to alleles may wane over time. Since haplotype diversity is maintained at an individual level, blood stage vaccines against polymorphic antigens present the challenge of how to overcome the diversity if immune responses are not cross-reactive and a multi-allelic design is taken as a most suitable approach.

## Supplementary Information

Additional file 1

Additional file 2

## Data Availability

Sequenced data have been deposited in the GenBank NIH genetic sequence database under accession numbers (OQ821998–OQ822147).
