# Supplementary figures and images for: Stable Plasmodium falciparum merozoite surface protein-1 allelic diversity despite decreasing parasitaemia in children with multiple malaria infections
Source: Malar J. 2025 Apr 28;24:136. doi: 10.1186/s12936-025-05378-7 (PMC12036178; doi:10.1186/s12936-025-05378-7)

# Supplementary figure 1

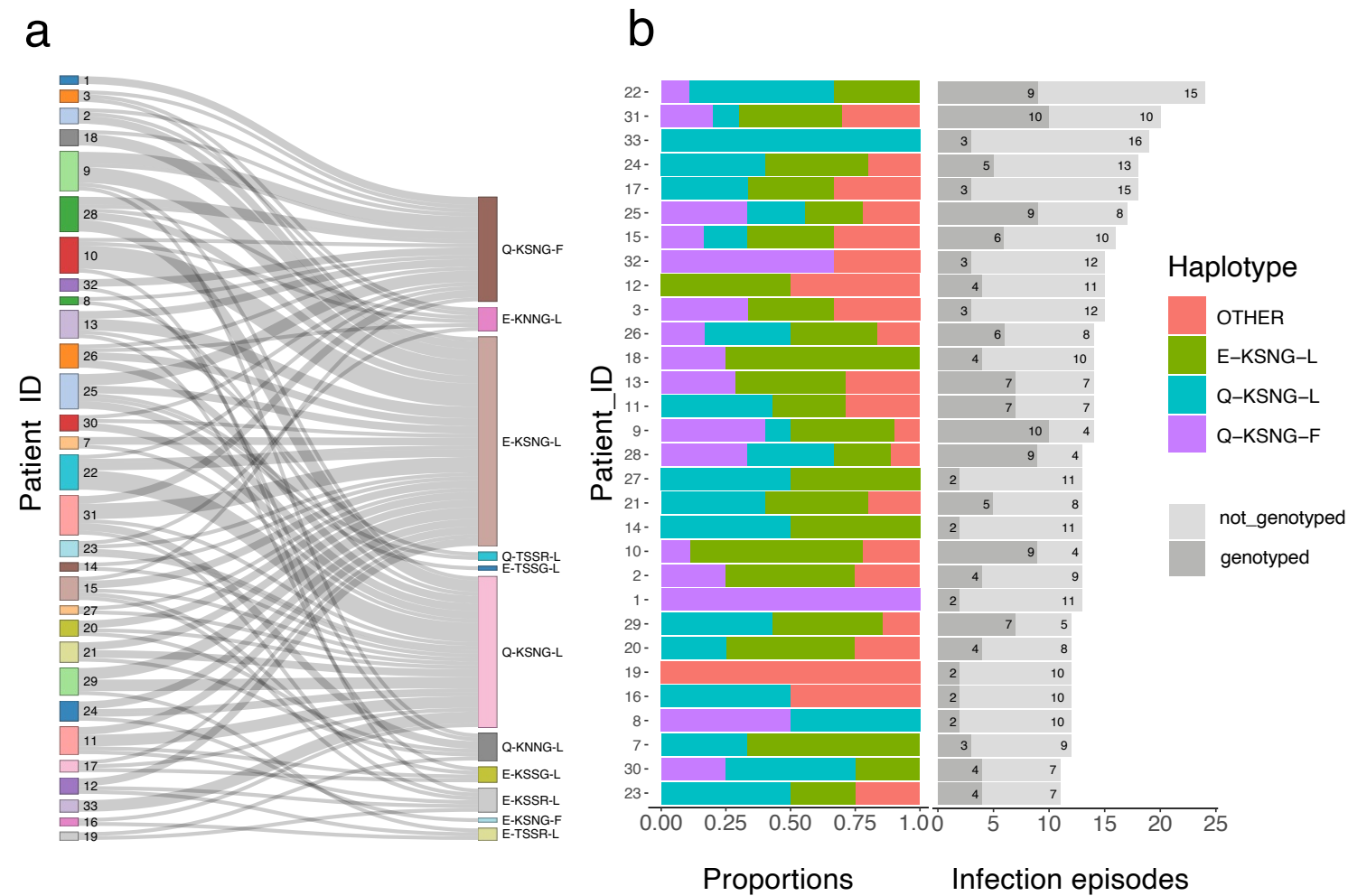

Supplement: Supplementary file 1 — Additional file 1 [file 12936_2025_5378_MOESM1_ESM.pdf]
